# Oral Microbiota Profile in a Group of Anti-AChR Antibody–Positive Myasthenia Gravis Patients

**DOI:** 10.3389/fneur.2022.938360

**Published:** 2022-07-07

**Authors:** Chao Huang, Feng Gao, Haitao Zhou, Li Zhang, Dandan Shang, Ying Ji, Zhihui Duan

**Affiliations:** ^1^Department of Neurology, Luoyang Central Hospital Affiliated to Zhengzhou University, Luoyang Cerebrovascular Disease (Stroke) Clinical Medical Research Center, Regional Medical Center for Neurological Diseases of Henan Province, Luoyang, China; ^2^Department of Neuroimmunology, Henan Institute of Medical and Pharmaceutical Sciences, Zhengzhou University, Zhengzhou, China; ^3^Department of Neurology, The Second Affiliated Hospital of Zhengzhou University, Zhengzhou, China

**Keywords:** oral microbiota, Myasthenia gravis, 16S rDNA sequencing, anti-AChR antibody, neuromuscular junction

## Abstract

Myasthenia gravis (MG) is an autoimmune disorder caused by autoantibodies directed against the postsynaptic membrane at the neuromuscular junction. Perturbation of gut microbiota is thought to contribute to the development of MG, as reflected by fecal metabolomic signatures in humans, but there have been few studies on the relationship between oral microbiota profile and MG. The current study evaluated the correlation between oral microbiota composition and diversity and anti-acetylcholinereceptor (AChR) antibody–positive MG by comparing oral microbiota communities of patients *(n* = 20) and healthy controls (HCs; *n* = 20) by 16S rRNA gene sequencing. Principal coordinate analysis and Adonis analysis revealed significant differences in oral microflora profile between the twogroups. Compared to HCs, the abundance of the phyla *Firmicutes* and *Actinobacteria* and genera *Streptococcus, Rothia*, and *Lachnoanerobaculum* was significantly increased whereas that of phyla *Proteobacteria* and *Spirochaetota*and genera *Neisseria, Haemophilus*, and *Treponema* was significantly decreased in MG patients. The Kyoto Encyclopedia of Genes and Genomes pathway analysis showed that the biosynthesis of ansamycins and amino acid metabolism pathways were altered in MG. These results indicate that oral microbiota composition is perturbed in patients with anti-AChR antibody–positive MG, providing new potential avenues for targeted therapeutic interventions.

## Introduction

Changes in gut microbiota composition can lead to immune-mediated diseases by affecting immune system activation (1, 2). Myasthenia gravis (MG) is an autoimmune disease caused by pathogenic autoantibodies targeting the neuromuscular junction (NMJ); these include anti-acetylcholinereceptor (AchR), anti–muscle-specific tyrosine kinase (MuSK), and anti–low-density lipoprotein receptorrelated protein 4 (LRP4) antibodies. Anti-AChR antibodyis detected with routine assays in 70–80% of all patients with MG. Based on the location of the affected muscles, MG is classified as ocular or generalized (3). The pathogenesis of MG is complex and involves an imbalance of gut microbiota (4, 5). Because most gut microorganisms originate in the oral cavity (6, 7), oral microbiota may also contribute to MG pathogenesis.

Poor oral health, which is associated with an imbalance of oral microbiota community structure and composition, has a major effect on neuroinflammation and has been linked to central nervous system diseases such as Alzheimer's disease (AD), to central nervous system diseases such as Alzheimer's disease (AD), Parkinson's disease (PD), migraine, and multiple sclerosis (MS) (6–10). However, the relationship between oral microbiota profile and MG has not been previously studied. To address this issue, in this study we performed 16S rDNA high-throughput sequencing of saliva samples from MG patients positive for antibody against acetylcholine receptor (AChR) and healthy controls (HCs) in order to evaluate oral microbiota diversity and abundance. The results provide new directions for research on the pathogenesis and treatment of MG.

## Materials and Methods

### Study Population

This study included 20 newly diagnosed MG patients (9 males and 11 females) positive for anti-AChR antibody who were admitted to Luoyang Central Hospital Affiliated to Zhengzhou University from January 2018 to January 2021. The average age (standard error [SE]) of the patients was 46.2 (15.5) years. Additionally, 20 HCs (12 males and eight females) without organic, systemic, or oral disease or family history of tumors were recruited from the general population; the average age (SE) of this group was 46.5 (14.6) years. There was no statistically significant difference in age or sex ratio between the twogroups. The study was approved by the ethics committee of Luoyang Central Hospital Affiliated to Zhengzhou University, and all patients signed an informed consent form.

Exclusion criteria were the use of antibiotics, glucocorticoids, probiotics, or antacids such as proton pump inhibitors that affect oral microbiota in the previous 30 days; any type of oral disease; chronic gastrointestinal diseases; history of gastrointestinal surgery; and pregnancy or lactation.

### Sample Collection and DNA Extraction

Subjects were required to refrain from eating, drinking, smoking, or chewing gum within 30 min before saliva sample collection. Unstimulated saliva was collected with salivettes and immediately placed on ice and stored at −80°C until use. DNA was extracted using the E.Z.N.A. Stool DNA Kit (Omega Bio-Tek, Norcross, GA, USA) according to the manufacturer's instructions and stored at −20°C until analysis. DNA integrity was evaluated by 1.2% agarose gel electrophoresis, and DNA concentration was measured with a NanoDrop spectrophotometer (Thermo Fisher Scientific, Waltham, MA, USA).

### PCR Amplification and Sequencing

The forward primer 5′CCTACGGGNGGCWGCAG-3′ and reverse primer 5′GACTACHVGGGTATCTAATCC-3′ targeting the hypervariable V3–V4 region (341F/805R) of the 16S rRNA gene was used for PCR amplification of the extracted DNA. The reaction was performed on a T100Thermal Cycler PCR system (Bio-Rad Laboratories, Hercules, CA, USA) with the following program: 3 min of denaturation at 95°C, followed by 21 cycles of 0.5 min at 94°C (denaturation), 0.5 min at 58°C (annealing), and 0.5 min at 72°C (elongation), with final extension at 72°C for 5 min. PCR products were detected by 1.5% agarose gel electrophoresis. Amplicons were purified using Hieff NGS DNA Selection Beads (YeasenBiotech, Shanghai, China) and quantified using the dsDNA HS Assay Kit for Qubit (Yeasen Biotech, Shanghai, China). According to the measured values, the product was mixed at equal ratios (50 ng) and sent to Shanghai Mobio Biomedical Technology Co. (Shanghai, China) for high-throughput sequencing on the Miseq platform (Illumina, San Diego, CA, USA) according to the standard manufacturer's protocols. The raw read data of all the samples were deposited in the National Center for Biotechnology Information BioProject database (https://www.ncbi.nlm.nih.gov/bioproject/, PRJNA837028).

### Bioinformatic Analysis and Statistical Analysis

Paired-end readswere spliced into a sequence, and read quality was evaluated by quality control filtering. Operational taxonomic units (OTUs) were classified based on 97% similarity after chimeric sequences were removed using UPARSE v7.1 (http://drive5.com/uparse/), and were annotated using the SILVA reference database (SSU138). Alpha diversity metrics (ACE estimator, Chao 1 estimator, Shannon–Wiener diversity index, and Simpson diversity index) were assessed using Mothur v1.42.1. Bray–Curtis weighted and unweighted UniFrac dissimilarity was calculated in QIIME (http://qiime.org/). Principal coordinate analysis (PCoA) plots and permutational multivariate analysis of variancewere used to test for statistical significance between groups with 10,000 permutations usingthe R v3.6.0 vegan 2.5-7 package. The linear discriminant analysis (LDA) effect size (LEfSe) was used to detect taxa with differential abundance in the twogroups (lefse 1.1, https://github.com/SegataLab/lefse). Random forest models were used to identify key discriminatory OTUs between groups. PICRUSt2 v2.4.1 (https://github.com/picrust/picrust2/wiki) was used to predict functional abundance based on 16S rRNA gene sequences. The nonparametric Mann–Whitney U test was used to evaluate significant differences between groups. All data were analyzed using SPSS v20.0 software (IBM, Armonk, NY, USA), and P<0.05 was considered statistically significant.

## Results

### Characteristics of the Study Participants

A total of 40 saliva samples were prospectively collected from 20 anti-AChR antibody-positive MG patients and 20 age- and sex-matched HCs. The clinical subtype was classified according to Myasthenia Gravis Foundation of America criteria. Clinical features and treatment information were obtained from patients' clinical records (Table 1).

**Table 1:** Clinical characteristics of the study subjects.

**Characteristic**	**MG**	**HC**	**P-value^†^**
Sample size	20	20	–
Female	11 (55%)	8 (40%)	0.342
Age, years	46.2 ± 15.5	46.5 ± 14.6	0.958
Thymoma/thymic hyperplasia	5 (25%)	–	–
Thymectomy	2 (10%)	–	–
Immunosuppressive treatment	6 (30%)	–	–
MGFA classification		–	–
I	12 (60%)	–	–
IIa	2 (10%)	–	–
IIb	6 (30%)	–	–

*Values are expressed as the mean ± standard error of the mean*.

† *Calculated with the 2-tailed Student's t-test for continuous variables (age) and χ^2^ test for categorical variables (sex)*.

*HC, healthy control subjects; MG, Myasthenia gravis patients; MGFA, Myasthenia Gravis Foundation of America*.

### Microbial Diversity in the MG and HC Groups

**Figure 1:**
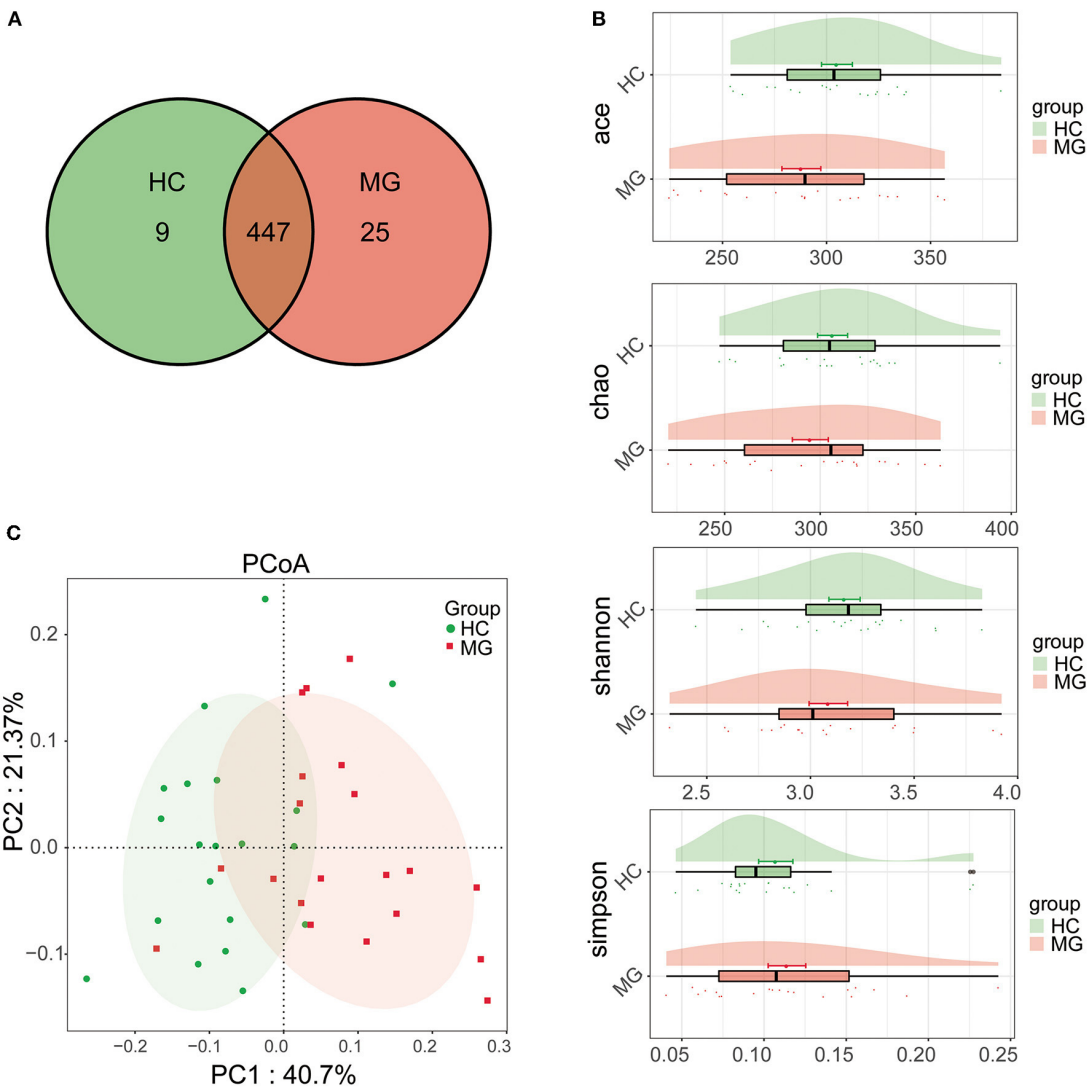
Comparison of microbial diversity between MG *(N* = 20) and HC (*N* = 20) groups. **(A)** Venn diagram of OTU distributions **(B)** Alpha diversity estimated by the ACE, Chao, Shannon, and Simpson indices **(C)** Beta diversity analyzed by PCoA based on a weightedUniFrac algorithm.

A total of 481 bacterial OTUs showing 97% similarity were detected in saliva samples including 472 OTUs in the MG group and 456 in the HC group. There were 447 OTUs shared by the two groups, 25 that were unique to MG patients, and 9 that were unique to HCs (Figure 1A). The alpha diversity of the oral microbiota in samples was compared based on ACE, Chao, Shannon, and Simpson indices; the diversity was slightly lower in the MG group than in the HC group, but the difference was not significant (Figure 1B). Beta diversity of oral microbiota was compared between groups by PCoA based on a weightedUniFrac algorithm. PC1 and PC2 represented overall flora by 40.7% and 21.37%, respectively. The MG and HC samples showed a trend of separation, indicating that there were differences in overall oral microbiota composition between the two groups (Figure 1C); this was corroborated by the results of the Adonis analysis (*P* < 0.001).

### Differences in Oral Microbiota Composition Between MG and HC Groups

**Figure 2:**
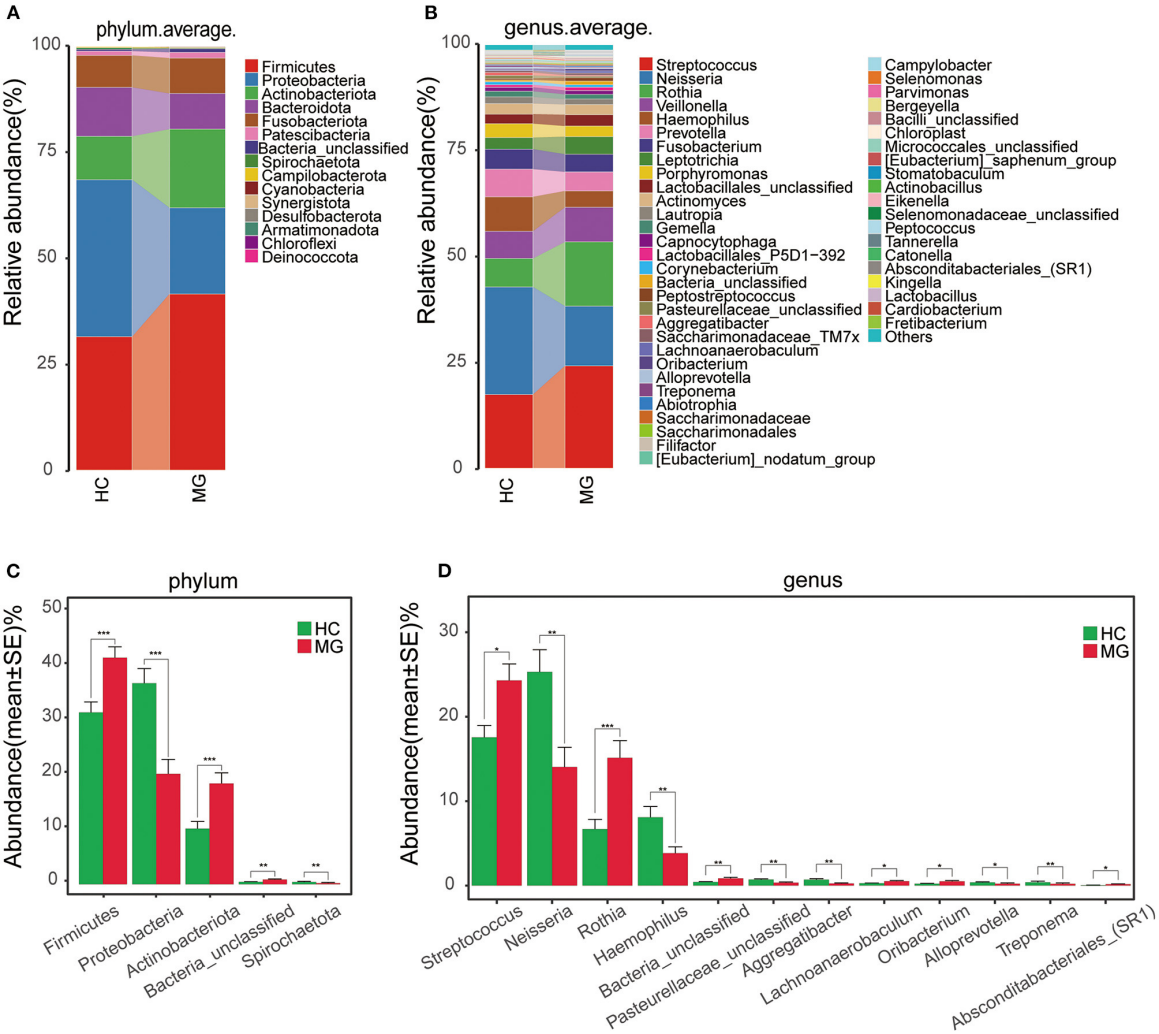
Composition and comparison of oralmicrobiomes in MG (*N* = 20) and HC (*N* = 20) groups **(A,B)** Composition of oral microbiota at the phylum **(A)** and genus **(B)** levels in MG vs. HC **(C,D)** Distribution of differential microbiota at the phylum **(C)** and genus **(D)** levels. Significant differences were observed in the abundance of predominant genera between MG (red) and HC (green) groups. The average abundance of each bacterial species is presented as mean±SE. *P*-values were calculated with the Mann–Whitney U-test.*P < 0.05; **P < 0.01; ***P < 0.001.

The average relative abundance of oral microbiota in the two groups was evaluated at the phylum and genus levels (Figures 2A,B). Firmicutes was the predominant phylum in the MGgroup, followed by Proteobacteria, Actinobacteriota, Bacteroidota, and Fusobacteriota. The oral microbiota community in the HC group was dominated by Proteobacteria, followed by Firmicutes, Bacteroidota, Actinobacteriota, and Fusobacteriota (Figure 2A). At the genus level, *Streptococcus* was the most abundant taxon in the MG group, followed by *Rothia, Neisseria*, and *Veillonella*; in the HC group, Neisseria was the most highly represented genus, followed by *Streptococcus, Haemophilus, Rothia, Veillonella*, and *Prevotella* (Figure 2B).

The Firmicutes and Actinobacteriota phyla were more abundantwhereas Proteobacteria and Spirochaetota were less abundant in the MG group than in the HCgroup (*P* < 0.05; Figure 2C). The *Streptococcus, Rothia, Lachnoanerobaculum*, and *Oribacterium*genera were significantly more abundant whereas*Neisseria, Haemophilus, Aggregatibacter, Alloprevotella*, and *Treponema*had lower abundance in the MG group than in the HC group (*P* < 0.05; Figure 2D).

**Figure 3:**
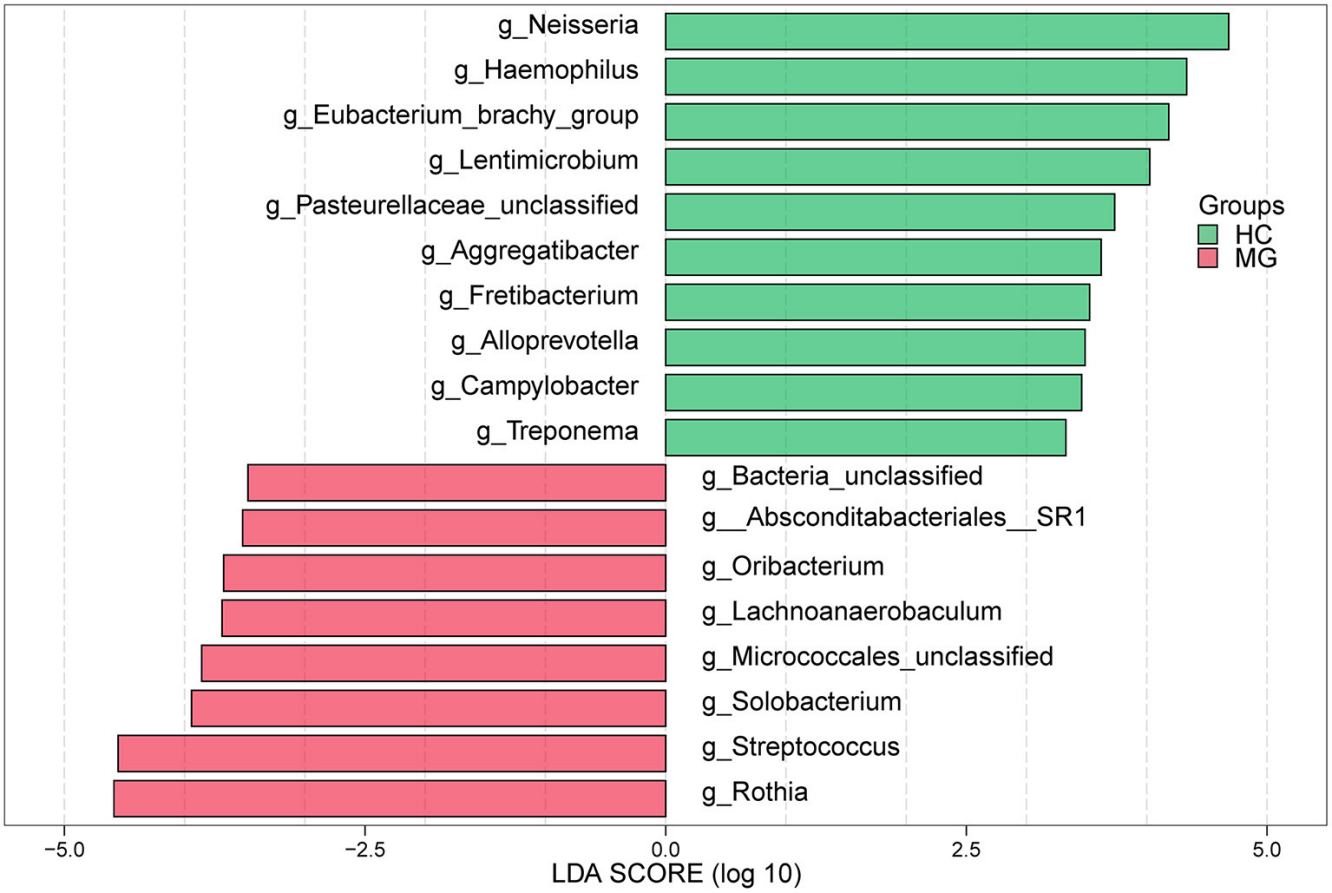
LEfSe analysis of microbial profiles between in MG (*N* = 20) and HC (*N* = 20) groups at the genus level. The histogram of LDA scores calculated for select taxa showed significant differences in microbe type and abundance between MG (green) and HC (red). LDA scores on a log_10_ scale are shown at the bottom. The significance of a microbial marker increased with LDA score.

In the LEfSe analysis, bacterial genera (LDA value >3) that differed significantly between the two groups were selected. The *Rothia, Streptococcus, Solobacterium, Lachnoanerobaculum*, and *Oribacterium*genera were more highly represented in the MG group whereas *Neisseria, Haemophilus, Eubacteriumbrachy, Lentimicrobium, Aggregatibacter, Fretibacterium, Alloprevotella, Campylobacter*, and *Treponema* were more abundant in the HC group (Figure 3), which was confirmed with the Mann–Whitney U test.

**Figure 4:**
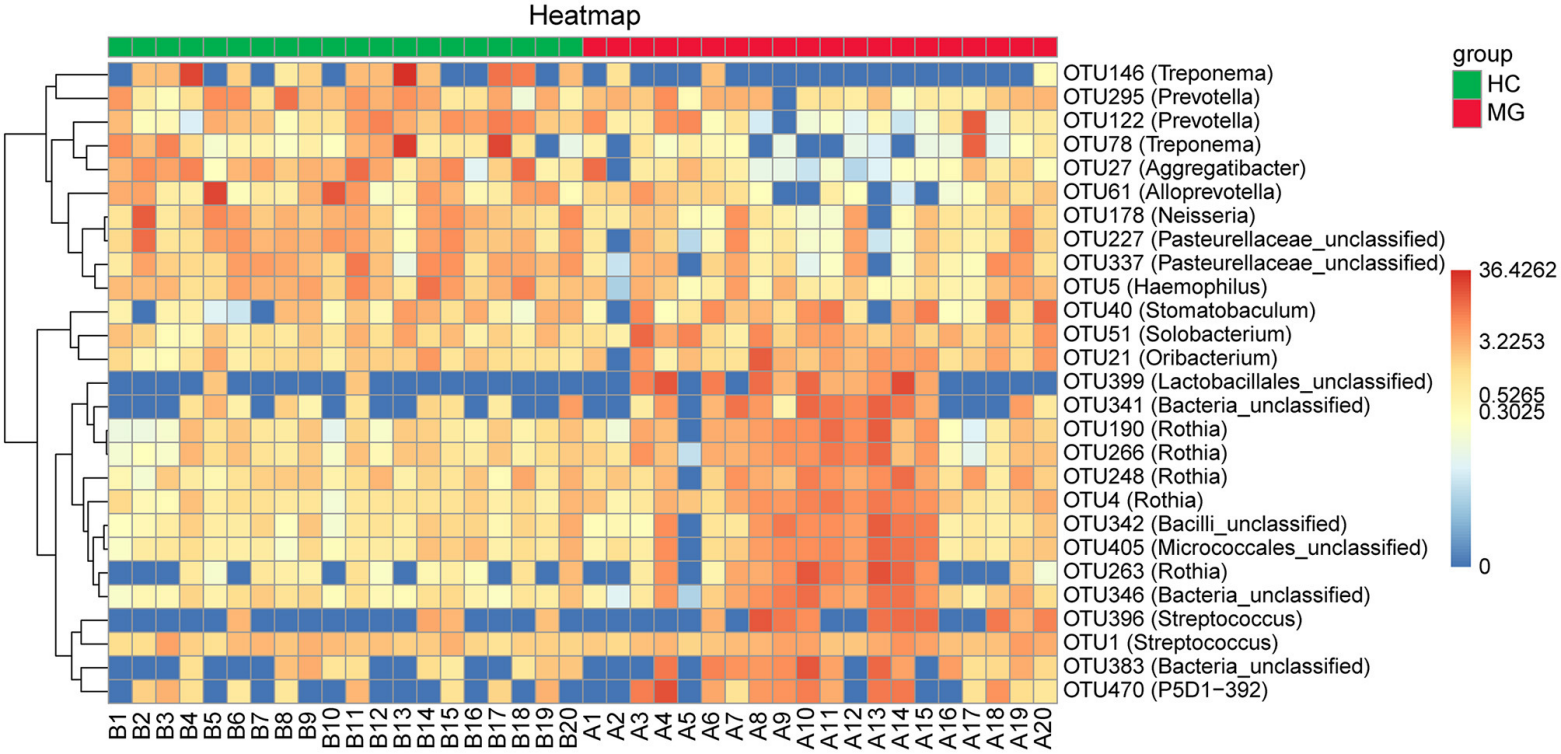
Heatmaps of the relative abundance of differential OTUs between MG (N=20) and HC (*N* = 20) groups. For each sample, the columns show relative abundance data for differential OTUs on the right. The relative abundance of each OTU was used to generate the heatmap (blue, low abundance; red, high abundance). Group data are shown above the plot: MG, left, right line; HC, right, blue line. Each row represents one OTU.

By randomforest analysis we found 27 OTUs that differed between the two groups; 17 (including the *Streptococcus, Rothia, Solobacterium, Stomatobaculum, Oribacterium*, and P5D1-392 genera) were significantly increased whereas 10 (including *Treponema, Prevotella, Alloprevotella, Neisseria, Aggregatibacter*, and *Haemophilus*) were significantly decreased in MG patients compared to HCs (Figure 4).

### Prediction of Gene Function

**Figure 5:**
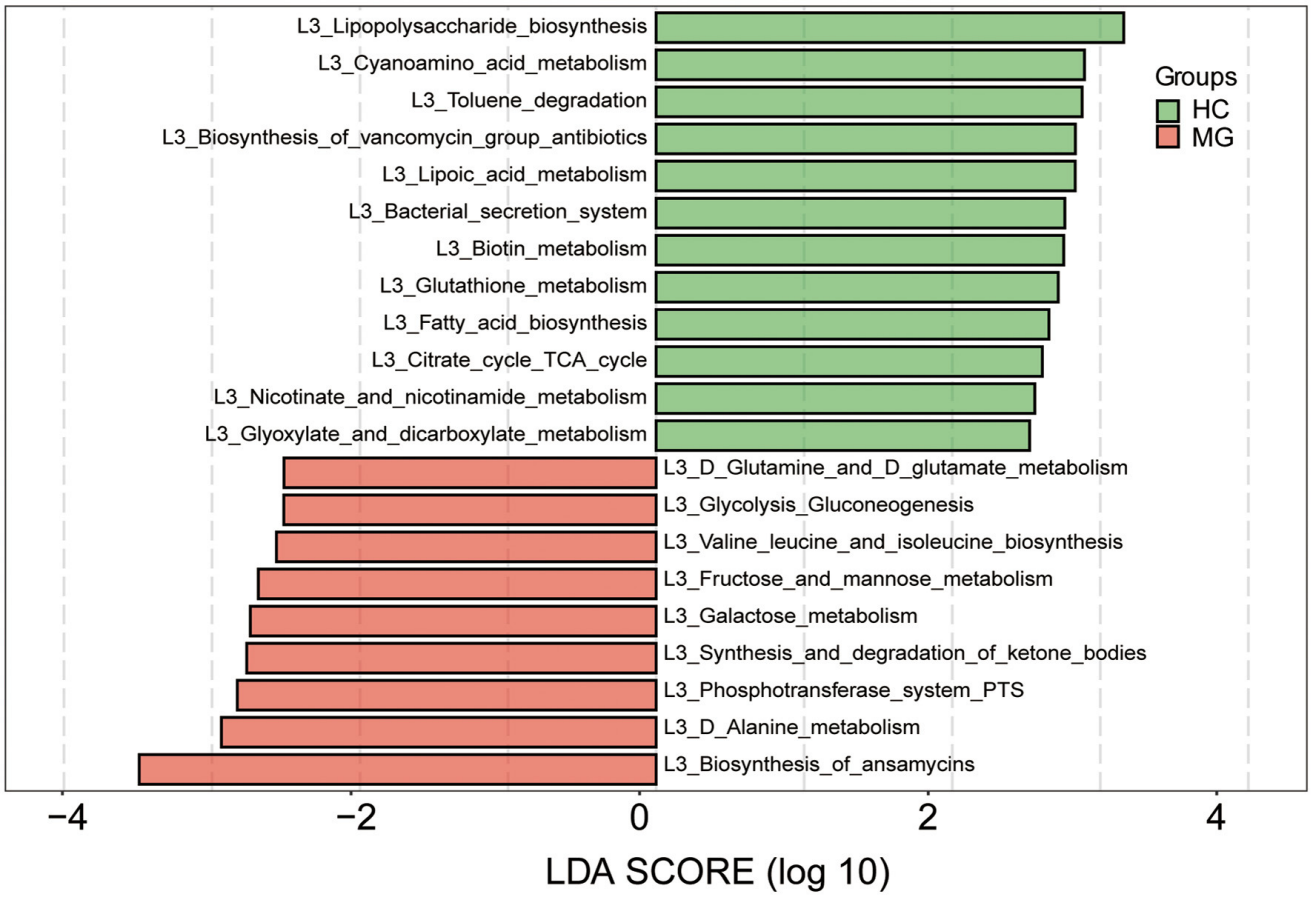
Functional analysis of predicted metagenomes. 16S sequencing data were used for function prediction based on KEGG Pathway databases (level 3), and LEfSe analysis was performed to selectmetabolic pathways (L3 level) with significant differences between MG (red) and HC (green) groups. LDA scores on a log_10_ scale are shown at the bottom. The significance of a microbial marker increased with LDA score.

We predicted the functions of the oral microbiota identified as being different between MG patients and HCs by Kyoto Encyclopedia of Genes and Genomes (KEGG) pathway analysis. KEGG pathways involved in biosynthesis of ansamycins, alanine metabolism, phosphotransferase system, synthesis and degradation of ketone bodies, galactose metabolism, fructose and mannose metabolism, valine leucine and isoleucine biosynthesis, glycolysis gluconeogenesis, and glutamine and glutamate metabolism were more highly represented in the MG group, whereaspathways involved in lipopolysaccharide biosynthesis, cyanoamino acid metabolism, toluene degradation, biosynthesis of vancomycin group antibiotics, lipoic acid metabolism, bacterial secretion system, biotin metabolism, glutathione metabolism, fatty acid biosynthesis, citrate cycle tricarboxylic acid cycle, nicotinate and nicotinamide metabolism, and glyoxylate and dicarboxylate metabolism were less represented compared to the HC group (LDA score ≥2.5, p < 0.05; Figure 5).

## Discussion

MG is an autoimmune disease characterized by variable muscle weakness, with the primary subtype attributed to antibodies binding to AChR at the NMJ (5, 11, 12). Forkhead box (Fox) P3+ cluster of differentiation (CD) 4+ regulatory T cells (Tregs) play a critical role in maintaining self-tolerance and immune homeostasis to prevent the development of MG (12); these cells are depleted in MG patients, which may contribute to the pathogenesis of this disorder (13, 14). The abundance of FoxP3+CD4+ Treg cells in the colonic lamina propria isinfluenced by the composition of gut microbiota. Intestinal flora composition is known to be imbalanced in MG. In stool samples from anti-AChR antibody-positive MG patients, the relative abundance of phylum Bacteroidetes was increased whereas that of Verrucobacterium and Actinomycetes was decreased compared to HC subjects (15); and another study showed that Proteobacteria and Bacteroidetes were enriched whereas Firmicutes abundance was reduced in MG. At the genus level, *Streptococcus* was shown to be more abundant whereas *Clostridium* and *Eubacterium*had lower abundance in MG patients compared to HCs (4). However, there have been no studies examining the oral microbiota profile in MG.

Saliva constitutes the external environment of the oral cavity and is an important biological sample for the study of oral microbiota, which is a major research focus of the National Institutes of Health Human Microbiome Project (16, 17). An imbalance of oral microbiota can not only cause various oral diseases but is also closely related to the development of tumors, diabetes, rheumatoid arthritis, cardiovascular diseases, and nervous system diseases (6). Changes in microflora community composition can lead to immune-mediated diseases by affecting immune system activation (1). Saliva is easy to collect and store and different collection methods have little effect on the quality and yield of extracted DNA. In this study, we used a non-invasive method to collect saliva samples from study participants and 16S rRNA high-throughput sequencing was performed to analyze the abundance and composition of the oral microbiota in patients with anti-AChR antibody–positive MG and HCs.

The results of this study showed that Proteobacteria, Firmicutes, Bacteroidota,Actinobacteriota, and Fusobacteriota were dominant taxa in the HC group. An analysis of the community structure of saliva microbial flora in healthy Han Chinese adults in Shanghai showed that the Firmicutes, Proteobacteria, Bacteroidota, Actinobacteriota, and Fusobacteriota phyla accounted for 97.54% of microorganisms in saliva (18). This is supported by our observations, indicating that the high-throughput sequencing results were reliable. The comparative analysis between MG patients and HC subjects revealed that the abundance of Firmicutes and Actinobacteriota was increased whereas that of Proteobacteria and Spirochaetota was decreased in the MG group compared to HCs. The Firmicutes/Bacteroidetes (F/B) ratio is widely considered to influence the maintenance of normal intestinal homeostasis (19); a higher ratio is associated with metabolic diseases such as steatosis and obesity (20), while a lower ratio has been linked to inflammatory diseases such as inflammatory bowel disease and Crohn's disease (19). The F/B ratio reflects a proinflammatory environment in which certain microbiota can cause damage to the intestinal epithelium, triggering an immune response that contributes to immunologic imbalance in autoimmune disorders. A previous study of gut microbiota showed that the F/B ratiowas significantly lower in MG patients than in HCs (4), but in our study, the abundance of Firmicutes was elevated in MG patients; this may be related to the difference in distribution of intestinal and oral flora, which warrants further investigation.

At the genus level, *Streptococcus, Rothia, Lachnoanerobaculum*, and *Oribacterium*were increased whereas *Neisseria, Haemophilus, Aggregatibacter, Alloprevotella*, and *Treponema*were decreased in MG patients compared to HC subjects. It was previously reported that the oral microbiome of MG patients had a higher abundance of *Streptococcus*, consistent with the profile of intestinal flora (4). The relative abundance of *Streptococcus* can alter the balance of the immune system through regulation of factors such as peroxisome proliferator activated receptor (PPAR) γ (21, 22). *Streptococcus* regulates PPARγ and its ligand 15D-PGJ2 by activating PPARγ and inhibiting certain pathways or immune cell functions. PPARγ is involved in regulating the proliferation and differentiation of immune cells including FoxP3+ CD4+ Treg cells (17), which maintain self-tolerance and immune homeostasis and play a key role in the occurrence of MG (13, 23, 24). *Rothia* species are opportunistic pathogens associated with various infections in immunocompromised and immunocompetent individuals. *Rothia* antigens can activate lymphocytesin patients with periodontal disease, and induce tumor necrosis factor α (TNF-α) production in macrophages. TNF-α is an important regulator of immunity and inflammation that promotes thymocyte activation and proliferation (25, 26). Some studies have shown that TNF-α level is significantly elevated in MG patients compared to normal subjects. TNF-α can directly destroy AChR or induce the differentiation and growth of B cells, thereby increasing AChR antibody production (27, 28). We speculate that changes in *Streptococcus*and *Rothia* abundance are involved in the development and progression of MG. Some oral microbiota that showed differential abundance between MG and HC groups in this study were different from those in intestine, which may be related to factors such as the small sample size, dietary habits, and environmental factors in different regions.

Salivary microorganisms prevent the invasion of pathogens by competing for nutrients in the oral cavity; they also participate in nutrient metabolism, promote oral immune system development, and interact to maintain the balance of the oral microenvironment (15, 29). An imbalance in oral microbiota community structure has been linked to neurologic diseases. For example, *Lactobacillus* and *Streptococcus*levels were significantly increased in the saliva of patients with AD (8). Oral flora may migrate to the brain and induce a neuroimmune response. One study reported significant differences in the relative abundance of 23 genera of oral bacteria in migraine patients compared tohealthy subjects, and it was proposed that they contribute to the occurrence of migraine via the nitrate–nitrite–nitric oxide pathway (9). Oral microorganisms can affect the inflammatory process of multiple sclerosis (MS), and controlling oral infection may slow MS progression (10). Our results showed that oral microbiota community composition was altered in patients with MG. Differences in functional pathways were also observed between MG patients and HC subjects based on KEGG pathway analysis: the biosynthesis of ansamycins; alanine metabolism; valine, leucine, and isoleucine biosynthesis; glutamine and glutamate metabolism; and other metabolic pathways were more highly represented in the MG group. However, it remains to be determined how these changes in metabolism contribute to the onset of MG. We propose the following as possibilities worth exploring. (i). Ansamycin is a macrolide; these molecules interfere with neuromuscular transmission, resulting in exacerbation or unmasking of MG symptoms (30). Additionally, ansamycin has antiproliferative and antineuroinflammatory effects (31) that may be involved in the pathogenesis of MG. (ii). MG is associated with perturbation of serum phenylalanine and tyrosine metabolism (32); in our study, we found that amino acid metabolism was altered in the MG group. As some of metabolites have immune-modulating properties, further analyses to integrate oral and serum metabolite changes is required to clarify the role of microbial metabolites in MG onset.

There were limitations to the current study. (i). Because MG patients and HCs were recruited at a single center, the sample sizes were small. The small sample size limited our ability to analyze potential confounding factors; therefore, the discriminatory power of the biomarker panel should be validated using samples from multicenter cohorts. (ii). We focused on a specific subset of MG patients—i.e., anti–AChR antibody-positive MG cases. Because most of these patients were mildly affected, our results may be biased. Longitudinal studies with larger samples that include different types of MG are needed to confirm our results. (iii). We showed that dysbiosis exists in MG, but it is unclear whether this is a cause and/or consequence of the disease; future studies should investigate the relationship between them.

To date, few studies have examined the correlation between oral microbiota and MG. Our study showed that the composition of the oral microbiota community was altered in anti-AChR antibody–positive MG patients, and suggested potential functional implication thereof. These findings provide a basis for the development of new strategies for the treatment of MG through modulationof oral microbiota.

## Data Availability Statement

The datasets presented in this study can be found in online repositories. The name of the repository and accession number can be found below: National Center for Biotechnology Information (NCBI) BioProject, https://www.ncbi.nlm.nih.gov/bioproject/PRJNA837028.

## Ethics Statement

The studies involving human participants were reviewed and approved by the Ethics Committee of Luoyang Central Hospital. Written informed consent to participate in this study was provided by the participants' legal guardian/next of kin.

## Author Contributions

ZD and CH designed the study. LZ, DS, and YJ collected clinical samples and performed the experiments. HZ analyzed the data. CH wrote the manuscript. FG and HZ revised the manuscript. All authors contributed to the article and approved the submitted version.

## Funding

This work was supported by the Medical Science and Technology Project of Henan Province (LHGJ20191201).

## Conflict of Interest

The authors declare that the research was conducted in the absence of any commercial or financial relationships that could be construed as a potential conflict of interest.

## Publisher's Note

All claims expressed in this article are solely those of the authors and do not necessarily represent those of their affiliated organizations, or those of the publisher, the editors and the reviewers. Any product that may be evaluated in this article, or claim that may be made by its manufacturer, is not guaranteed or endorsed by the publisher.
